# Expansion of antibody secreting cells and modulation of neutralizing antibody activity in HIV infected individuals undergoing structured treatment interruptions

**DOI:** 10.1186/1479-5876-11-48

**Published:** 2013-02-22

**Authors:** Anuska Llano, Jorge Carrillo, Beatriz Mothe, Lidia Ruiz, Silvia Marfil, Elisabet García, Eloísa Yuste, Víctor Sánchez, Bonaventura Clotet, Julià Blanco, Christian Brander

**Affiliations:** 1 Irsicaixa AIDS Research Institute – HIVACAT, Hospital Universitari Germans Trias y Pujol, Badalona, Spain; 2 Fundacio Lluita contra la SIDA, Hospital Universitari Germans Trias y Pujol, Badalona, Spain; 3 Universitat Autònoma de Barcelona, Hospital Universitari Germans Trias y Pujol, Badalona, Spain; 4 Retrovirology and Viral Immunopathology Laboratory, IDIBAPS, HIVACAT, Hospital Clínic, University of Barcelona, Barcelona, Spain; 5 Institut d’Investigació en Ciencies de la Salut Germans Trias i Pujol, IGTP, Badalona, Spain; 6 Institució Catalana de Reserca y Estudis Avançats (ICREA), Barcelona, Spain

**Keywords:** ASC, Neutralization activity, HIV-1, STI, IgG

## Abstract

**Background:**

HIV-1 infection generates numerous abnormalities in the B cell compartment which can be partly reversed by antiretroviral therapy. Our aim was to evaluate the effects that re-exposure to HIV antigens might have on the frequency and functionality of antibody secreting cells (ASC) in patients undergoing structured treatment interruptions (STI). As re-exposure to viral antigens may also boost the production of (neutralizing) antibodies, we also assessed the neutralizing activities during STI cycles.

**Methods:**

Retrospective study of 10 patients undergoing 3 cycles of STI with 2 weeks on and 4 weeks off HAART. ASC frequencies were determined by flow cytometry in samples obtained at the beginning and the end of STI. Neutralization capacity, total IgG concentration and anti-gp120-IgG titres were evaluated.

**Results:**

As expected, median viral loads were higher at the end of STI compared to on-HAART time points. The level of CD27 and CD38 expressing ACS followed the same pattern; with ASC being elevated up to 16 fold in some patients (median increase of 3.5% ± 4.13). Eight out of 10 patients maintained stable total IgG levels during the study. After purifying IgG fractions from plasma, HIV-neutralizing activity was observed in the two subjects with highest anti-gp120 titers. In one of these patients the neutralizing activity remained constant while the other showed elevated neutralizing Ab after first STI and once treatment was reinitiated after the 2^nd^ STI.

**Conclusions:**

Our data suggest that STI and its associated transient increases in viral load drive the frequencies of ASC in an antigen-specific manner. In some subjects, this re-exposure to autologous virus boosts the presence of neutralizing antibodies, similar to what is seen after influenza vaccination. STI may not boost clinically beneficial nAb levels but offers opportunities to isolate nAb producing cells at considerably higher levels than in subjects with completely suppressed viral replication.

## Background

Clinical parameters to assess HIV disease progression focus largely on CD4 T cell counts and CD4/CD8 T cell ratio, yet the B cell compartment of HIV infected subjects can be severely impacted by chronic HIV infection as well. This includes hypergammaglobulinaemia [1,2] and many other alterations in different B cell subpopulations, such as increased frequencies of immature transitional B cells, expansion of activated mature B cells, increased levels of memory B cells with an exhausted phenotype and a pronounced loss of resting memory B cells [3-8]. Many of these defects appear to be at least partly reversible by antiretroviral treatment and subsequent reduction in antigen viral loads as declines in hypergammaglobulinaemia, reductions in the number of HIV-1-specific and non-specific B cells and the normalization of other B cell subpopulations have been observed after starting HAART [5,9]. However, the recovery of memory resting B cells is incomplete despite prolonged times on HAART and sustained viral suppression [10,11], leading to a marked deficiency in rapidly expanding antigen-specific B cells and differentiation into antibody secreting cells (ASC) after re-exposure to the cognate antigen [12]. While extensive work has been conducted in isolating and characterizing the products of these cells (i.e. neutralizing antibodies), less is known about their fate in chronic HIV infection and after vaccine boosts. Doria-Rose et al. have reported that the frequency of ASCs was higher in HIV-infected patients than in uninfected donors [13] and a study by Moir et al. has shown that the peripheral blood B cell populations is especially enriched in plasmablasts and resting memory B cell in acute and early stages of HIV infection compared to chronically HIV infected individuals [14]. These differences were maintained after one year on HAART of both early and chronically HIV-infected individuals and were associated with a better functional profile of memory B cells in early treated subjects.

There is currently a great interest in the field to elicit broadly neutralizing antibodies (NAb) by different HIV-1 vaccine regimens as well as to identify, isolate and characterize novel monoclonal nAb. There are also encouraging results from the RV114 phase III vaccine trial, which has associated IgG responses against the V1-V2 region of gp120 and specific viral mutations in these regions with relative control from infection [15-17]. However, similar to previous human immunizations with envelope monomers or trimmers, no (broadly) neutralizing antibodies were induced. In the best of the cases, earlier studies showed vaccine-induced antibodies able to neutralize easily neutralizable viral isolates or the vaccine strain only [18-21]. These limitations highlight the need for the design of novel immunogens to elicit broadly neutralizing antibodies, although difficulties related to masked epitopes or tolerance mechanisms induced by self-homologies, among others, remain [22-24]. Understanding the nature of virus-specific ASC, their clonal composition and their induction upon antigen exposure may help to overcome some of these hurdles and provide important guidance of how such responses could be induced in vivo.

In an elegant report studying the expansion of Influenza-virus specific ASC and their neutralizing Ab production, Wilson and colleagues used Flu vaccination to re-expose and restimulate the Flu-specific B cell memory in vivo. Their results showed a rapid, virus-specific expansion of IgG^+^ plasmablastic B cell (i.e. CD27/CD38-expressing, antibody secreting cells) that peaked at day 7 after vaccination and which could reach up to 6% of all peripheral B cells. This significant expansion allowed them to isolate and characterize more than 50 novel nAb that were able to bind with high affinity to several Flu vaccine strains. We here followed the same rational and hypothesized that HIV infected individuals undergoing structured HAART treatment interruption would be similarly exposed to viral antigen and may boost at least transiently their HIV-specific ASC response. These expectations were supported by data showing unusually high titers of neutralizing antibodies in patients who were intermittently adherent to therapy and showed brief viremic periods as well as by earlier studies by Montefiori et al., where neutralizing antibodies emerged after treatment interruption [25-27]. For the present study, we studied 10 HIV-1 chronic infected patients who underwent 3 serial structured treatment interruptions (STIs) and tested whether physiological HIV-antigen re-exposure would similarly lead to alterations in their ASC populations and their HIV neutralization capacity.

## Methods

### Ethics statements

The study was approved by the ethics committee of the Hospital Germans Trias i Pujol. All patients provided written informed consent for the initial “AUTOVAC-2” study, which covered further retrospective analyses on stored samples.

### Patient selection

Ten subjects were selected among a larger cohort of fifty HIV-1 positive individuals who were enrolled in a past, randomized trial (AUTOVAC-2) that aimed to study the relative benefits of an on/off HAART regimen on viral control. The STI in this trial consisted of 6 consecutive cycles of two weeks off treatment followed by 4 weeks on treatment. The actual duration of periods without treatment differed among participants as treatment had to be reinitiated if viral rebound exceeded 10,000 HIV-1 RNA copies/ml. All individuals fulfilled the following inclusion criteria: having had a viral load (VL) < 50 copies/ml for at least 2 years, a CD4/CD8 T cell ratio above 0.7 at least during 6 months before the study entry, nadir CD4 count above 400 cells/mm^3^ through the whole course of the disease and current treatment including protease inhibitors (PIs). The original trial included a control arm without STI (group A), a group B where STI were conducted without IL2 administration and a group C where IL-2 (0.5 mU/BID) was administrated on day 5 after treatment re-introduction for 5 days. For the present study, 5 individuals were selected from group B (no IL2) and group C (with IL2 after STI) based on sample (plasma, PBMC) availability for 3 (of the 6) consecutive STI cycles. Main clinical characteristics are shown in Table 1.

**Table 1:** Patient characteristics

**Patient Id**	**Age (years)**	**Sex**^**a**^	**Years since diagnosis**	**Baseline VL**^**c **^**(RNA copies/ml)**	**Baseline CD4 cell count (cells/mm**^**3**^**)**	**Baseline CD8 cell count (cells/mm**^**3**^**)**	**Baseline treatment**^**b**^	**IL-2 administration**
1	42	F	5	80	1327	1225	D4T + 3TC + NFV	NO
2	54	M	12	33	801	763	D4T + 3TC + NFV	NO
3	39	M	2	50	580	548	DDI + 3TC + NFV	NO
4	34	M	15	1200	672	819	ABV + DDI + EFV	NO
5	41	F	8	20	2702	1061	3TC + NFV + AZT	NO
6	45	F	7	20	1548	1380	DDI + D4T + NFV	YES
7	51	F	16	20	956	697	D4T + IDV + 3TC + RTV	YES
8	55	M	4	20	801	744	D4T,3TC,NFV	YES
9	61	M	5	20	1122	570	IDV + 3TC + AZT	YES
10	51	M	9	20	987	810	EFV + D4T + 3TC	YES

^a^M: male; F: female.

^b^D4T: Stavudine; 3TC:Lamivudine; NFV: Nelfinavir; DDI: Didanosine; ABV: Abacavir; EFV: Efavirenz; AZT: Zidovudina; IDV: Indinavir; RTV: Ritonavir.

^c^ In patient 4 VL value at baseline of 1200 correspond to a blip in viral load as, both prior and post values are < 20.

### ASC identification

The phenotype and the frequency of ASC were determined by flow cytometry following a previously described gating strategy [13], using frozen PBMC samples and the following monoclonal antibodies conjugated with the indicated fluorochrome: CD3-allophycocyanin (APC), CD19-fluores-cein isothiocyanate (FITC), CD27-phycoeritrin-cyanine7 dye (PE-Cy7) and CD38-phycoeritrin (PE) (BD, Barcelona, Spain). Samples were acquired on an LSRII cytometer and data analysis was done using the FACSDiva Software v6.1.3 (BD, Barcelona, Spain). Briefly, lymphocytes were identified by forward and side scatter and B cells gated as CD3 negative, CD19 positive cells. ASC were identified among CD19^+^ population as those cells with high expression of both CD27 and CD38.

### Anti-gp120 IgG determination

The IgG response against gp120 was evaluated by ELISA as previously described [28] with few modifications. Briefly, recombinant gp120 (NIH AIDS Reference and Reagent Program) was adsorbed overnight at 4°C on a 96-well Maxisorb plate (Nunc). Then, the plate was blocked 3 hours at room temperature with 300 ul/well of blocking buffer containing PBS, 10% fetal bovine serum (Invitrogen) and 0.05% tween20 (Sigma-Aldrich). After washing, serial dilutions of the plasma samples, also in blocking buffer, were incubated overnight at 4°C. Serial dilutions of the IgGb12 antibody (Polymun) in blocking buffer were used as standard. The wells were washed again and 100 ul/well of a HRP-F(ab’)_2_ goat anti-human IgG Fc specific (Jackson-Immunoresearch), diluted 1/25,000 in blocking buffer, was added and incubated during one hour at room temperature. Finally, the wells were washed and the chromogenic reaction developed using OPD substrate and stopped with 4 N sulphuric acid. Absorbance at 490/620 nm was measured.

### Total IgG quantification

The total IgG quantification of plasma samples was performed by ELISA. Multiabsorbed and purified goat anti-human IgG-Fc specific F(ab’)_2_ (Jackson-Immunoresearch) was used as capture antibody. This antibody was added in 50 μl at 1ug/mL in PBS to each well of a 96-well Maxisorb plate (Nunc) and incubated overnight at 4°C. After washing and blocking, plasma samples, diluted in blocking buffer (see above), were added and incubated during 2 hours at room temperature. As standard, serial dilutions of a control plasma pre-quantified by nephelometry was used. After washing the secondary antibody (see above) was added and incubated for one more hour. Finally, the chromogenic reaction was performed as described previously [28].

### Neutralization assay

Plasma samples, inactivated for 1 hour at 56°C, and purified IgGs were used to evaluate virus-specific neutralization activity, using the TZM-bl (NIH AIDS Research and Reference Reagent Program) neutralization assay previously described by Montefiori et al. [29]. Briefly, three-fold serial dilutions of plasma samples (starting at a 1/60 dilution) or purified IgGs (starting at 2 mg/mL) were incubated in 96-well plates in duplicates for 1 hour with 200TCID50 of two viral strains NL4-3 (NIH AIDS Research and Reference Reagent Program) and BAL (MRC AIDS Reagent Program), two clinical isolates AC10 (NIH AIDS Research and Reference Reagent Program) and SVBP16 and a vesicular stomatitis virus (VSV)-pseudotyped on a HIV NL4-3 viral backbone included as control for HIV-Env unspecific neutralization activity. 10^5^ TZM-bl cells were added per well and incubated for 48 hours at 37°C and 5% CO_2_. Luciferase activity was determined using the Bright-Glo Luciferase Assay System (Promega, Madrid, Spain) and the luminescence was then quantified in a luminometer (Fluoroskan Ascet FL, Labsystem). The relative light units (RLU) obtained were used to calculate the percentage of neutralization using the formula: % Neutralization = 1-(R-Rcc/Rvc) × 100; where R = RLU of the tested plasma, Rcc = RLU of cell alone and Rvc = RLU of virus alone. The 50% of the inhibitory dose ID_50_ was calculated.

### IgG purification

Immunoglobulin G was purified from selected samples using the Ab Spin Trap kit (GE Healthcare, Barcelona, Spain) according to manufacturer instructions. Briefly, 125 μl of inactivated plasma (1 hour at 56°C) were loaded into the protein G sepharose column and incubated for 30 min at room temperature, to bind IgG antibodies. The IgG-depleted plasma fraction was eluted and kept at −80°C. After extensive washing with PBS, IgGs were eluted with 0.1 M glycine buffer (pH = 2.8) into tubes containing neutralizing buffer (1 M TrisHCl pH8.5) to preserve the activity of acid-labile IgGs. The purified samples were then buffer-exchanged into PBS and concentrated using Amicon Ultra-0.5 filters (Millipore, Barcelona, Spain). Finally, IgG concentrations were determined by measuring the absorbance at 280 nm in a spectrophotometer (BioPhotometer, Eppendorf).

### Statistical analysis

ASC frequencies and VL levels were expressed as median values and compared between the three STI, both in the on-treatment and off-treatment samples using the Wilcoxon matched pairs test when comparing non-parametric paired data, or Mann–Whitney test when comparing non-parametric unpaired data. Differences were considered statistically significant if p < 0.05. Relationships between different variables were assessed using Spearman rank order non-parametric correlation. All the statistical analyses were performed with GraphPad Prism v4.

## Results

### Expansion of ASC frequencies after STIs are driven by HIV viral loads

**Figure 1:**
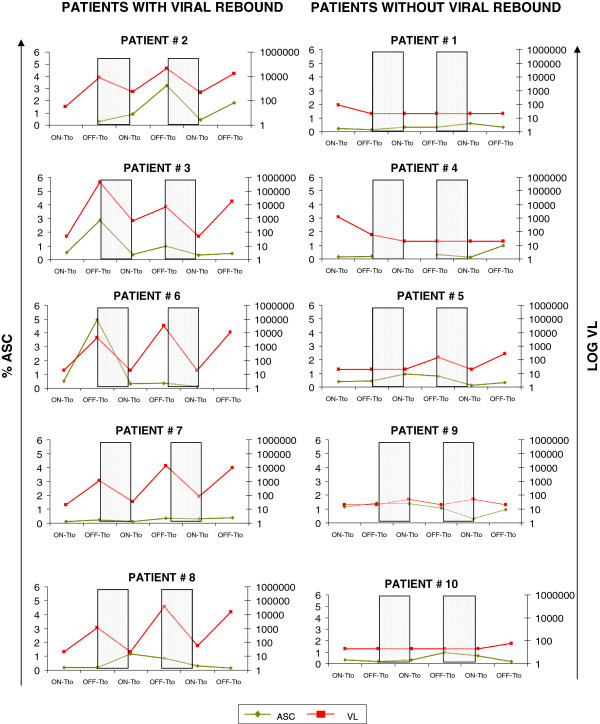
**The frequency of ASC was increased after treatment interruption.** ASC frequency (measured as % of ASC) and viral load (measured as Log10HIV-1RNAcopies/ml) dynamics along the study period for the five patients with viral rebound (left panels) and the five patients without viral rebound (right panels), after treatment interruption are shown. The on-treatment samples correspond to the time of stopping treatment in each STI, whereas the off-treatment sample correspond to the moment of treatment re-start after the correspondent STI. Intervals when subjects were on therapy are shaded.

In order to examine the effect of STI on ASC populations and its correlation with changes in HIV viral load, the frequencies of ASC in the peripheral blood were determined for 10 individuals undergoing 3 cycles of STI. ASCs were defined among CD19+ B cells as expressing high levels of CD27 and CD38 as described previously [13]. Samples were obtained on the day of treatment stop (“on-treatment”) and at the end of the interruption period (“off-treatment”) for each of the three STI. Five of the 10 patients: patients # 2, 3, 6, 7 and 8 (2 without IL2 [patients # 2 and 3] and 3 with IL2-treatment [patients # 6, 7 and 8], respectively) showed a rapid viral rebound after treatment interruption to at least 10^3^ viral copies/ml, whereas the remaining five individuals (patients # 1, 4, 5, 9 and 10) always maintained their viral loads to <290 viral copies/ml in all of the interruptions (Figure 1, the missing points in Patients #2, #4 and #6 for %ASC is due to lack of samples). The lack of viral rebound in those five patients 2 weeks after treatment interruptions is in line with a median time to viral rebound of 14 days seen in previously published trials assessing STI [30], and thus does not necessarily imply effective immune control of the virus in these 5 subjects. The addition of IL-2 therapy did not show a significant effect on viral control, neither in our selected 5 individuals nor in the overall cohort (with 8 out of 21 patients with IL-2 administration controlling viral rebounds after 2 weeks off treatment).

**Figure 2:**
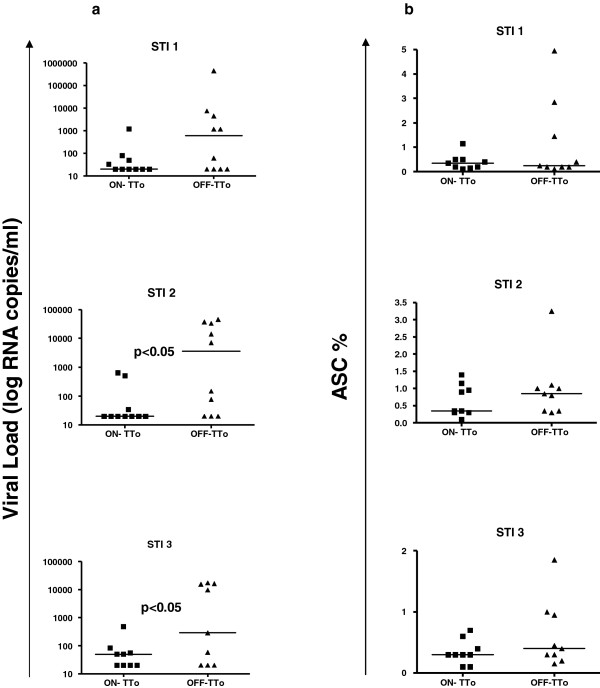
**Changes in Viral load and ASC frequency in the 3 STIs. **(**a**) Viral load and (**b**) ASC frequencies are higher in the post-treatment samples than in the pre-treatment ones after the 3 STI analyzed. The bars represent the median values for each group. Significant differences are indicated.

In order to examine whether active viral replication was driving the expansion of ASC, viral copy numbers per ml and ASC levels were compared between on- and off-treatment time points. As expected, median viral loads were always lower in the on-treatment samples than in the off-treatment time points: median <20 viral copies/ml [<20-1200] and 615 [<20-452,000] for the first STI, <20 [<20-654] and 3655 [<20-45900] (p = 0.015) for the second STI and <50 [<20-482] and 290 [<20-17400] (p = 0.031), respectively for the third STI (Figure 2a). Although the differences did not reach statistical significance, the same tendency was observed for the levels of peripheral blood ASC, with the median frequency of the ASC being lower in on-treatment samples than in samples from off-treatment time points after the second and the third STI: 0.35% ASC [0.10-1.15] versus 0.25% [0.10-4.95] for the first STI, 0.35 [0.10-1.40] vs 0.85 [0.30-3.2] for the second STI and 0.30 [0.10-0.70] vs 0.40 [0.15-1.85], respectively for the third STI (Figure 2b). Although insufficient PBMC were available to directly determine the HIV-specificity of ASC, the recent report by Doria-Rose et al. indicates that HIV-specific ASC are not infrequent [13]. However, it is likely that other specificities also contribute to the changes in total ASC frequency in the peripheral blood. Across the 10 individuals, the frequency of ASC among total peripheral blood B cells showed marked fluctuations over the serial STI, with all individuals increasing their ASC frequency at least 5–fold at some point during the three on/off HAART cycles (Figure 1). However, the most significant changes were observed for individuals who showed a viral rebound to >1100 viral copies/ml during any of the interruptions (Figure 1, left hand panels). Indeed, there is a trend (although no significant) for a direct correlation (r = 0.58; p = 0.08) between the maximal fold change in the percentage of ASC and the maximal value of viral load (data not shown). Although larger studies will be needed to achieve statistical power, these results suggest that increases in ASC could be driven by viral antigen load, particularly in subjects who showed strongest viral reactivation after treatment cessation. This potential association between increased viral loads and higher levels of ASC is further supported by the observation that both, median viral load and median frequency of ASC followed the same tendency over the course of the three STI: the median viral load and ASC frequency were higher after the second STI (3655 copies/mm^3^ [20–45900] and 0.82% ASC [0.30-3.25] respectively) than after the first STI (615 [20–452000] for viral load and 0.27% [0.10-4.95] for ASC, respectively), and lower median values are observed after the third STI (290 [20–17400] viral load and 0.40% [0.15-1.85] ACS, respectively) than after the second STI (data not shown). At the same time, the median percentage of total B cells remained remarkably stable (9.8% [4.1-14.6] after the first STI, 9.6% [1.6-19.15] after the second STI and 11.1% [5–14.6] after the third STI, respectively. This indicates that the changes in ASC were not due to a general B cell expansion but may reflect fluctuations in the HIV antigen specific B cell population. None of the above analyses showed significant differences when the patients were stratified based on IL2 administration (data not shown), in line with a lack of a clinical benefit of IL2 administration that was observed in the overall cohort

### STI boost HIV-specific IgG in a subset of patients

**Figure 3:**
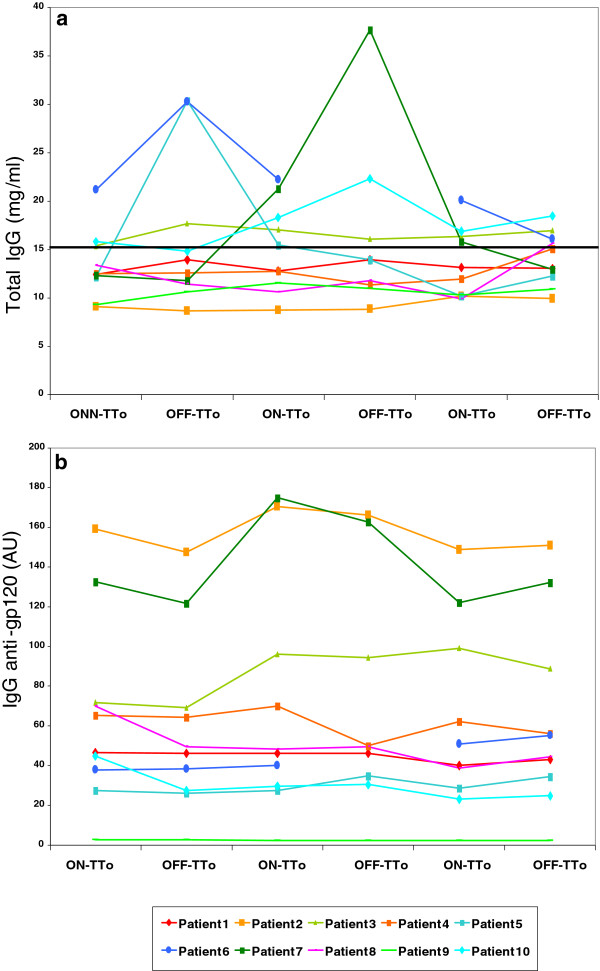
**Total IgG levels and anti-gp120 titers are shown for all patients along the study period. **(**a**) Total IgG levels with the normal cut-off set at 15 mg/ml and (**b**) anti-gp120 titers are shown.

**Figure 4:**
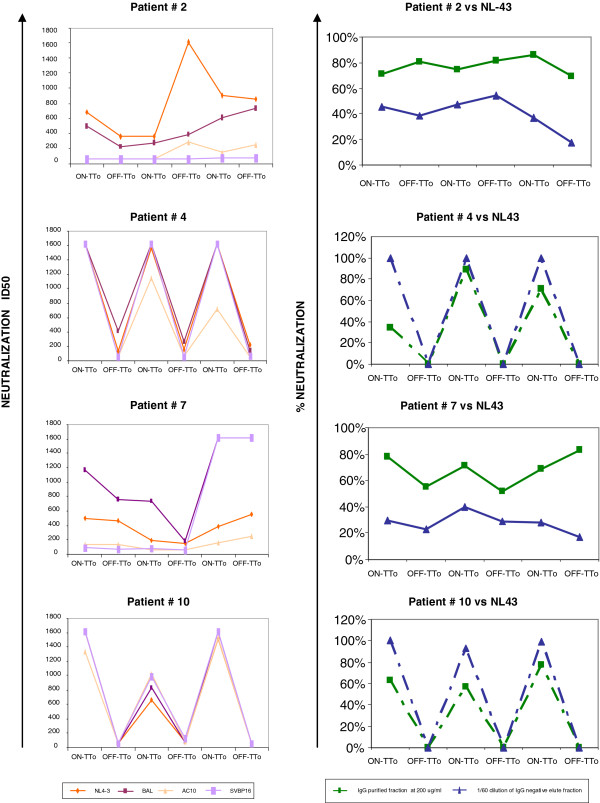
**Neutralization activity.** Neutralization activity (ID50) against NL4-3, BAL, AC10 and SVBP16 in inactivated plasma samples of 4 patients are depicted longitudinally (Left panels). The percentage of neutralization in the IgG purification fraction (200 μg/ml) and in the negative fraction (1/60 dilution) are longitudinally depicted for the 4 patients against NL4-3 (Right panels). In patients #4 and #10 dashed line represents theoretical union between the percentages of neutralization measured in the on-treatment samples with the non-measured ones in off-treatment samples.

As treatment interruptions did not affect the total peripheral B cell numbers and the observed changes in ASC levels are suggestive of a HIV antigen-specific B cell expansion, we next determined total IgG levels in serum samples from all patients at all time-points along the study period. At baseline (i.e. the first on-treatment sample in STI 1) seven out of ten patients had normal (<15 mg/ml) total IgG concentrations in plasma (median value in mg/ml 12.4 [10.7-21] whereas patients #3, #6 and #10 had elevated plasma IgG levels (>15 mg/ml, Figure 3a, the missing points in Patients #6, both in total IgG, Figure 3a, and in anti-gp120-specific IgG, Figure 3b, is due to lack of samples). Among the patients with normal IgG levels at baseline, two (patients #5 and #7) showed a transient increase in total IgG after the first and second STI, respectively, before reaching normal concentrations again during the third treatment interruption cycle. To monitor HIV-specific IgG levels, anti-gp120 titers were evaluated in plasma samples from the same time points. While anti-gp120 IgG levels were detectable in all subjects at baseline, patients #2 and #7 both maintained particularly high levels of anti-gp120 titers all along the study period (>100 AU IgG anti-gp120, Figure 3b). Of note, both patients showed highest levels of anti-gp120 IgG during the second STI, at times when their rebounding virus also reached maximal levels (Figure 4a and Figure 2). Together, the data indicate that the majority of the patients (7 out of 10, 70%) did not significantly increase their total IgG levels during STI cycles, suggesting that viral reactivation was not inducing a pronounced hypergammgobulianemia.

### Ig-mediated neutralization activity can be increased after STI

To assess whether increases in HIV-specific IgG titers during STI extend to neutralizing Ab as well, the production of neutralization antibodies (NAb) was determined in all 10 subjects for all three STI cycles. Neutralization assays were carried out using two laboratory viral strains NL4-3 and BAL and two clinical viral isolates (AC10 and SVBP16) in the TZM-bl cell line based neutralization assay [29]. Five out of ten patients presented low or no neutralization activity (<200 ID_50_ value) and one patient presented median neutralization activity (200–1000 ID_50_ value) (data not shown). The remaining four patients had high neutralization titers (>1000 ID_50_ value) in at least one of the samples tested (Figure 4, left hand panels). Of note, those patients were also the ones infected for longest (Table 1, 9–16 years, p = 0.0095), in accordance with previous publications from several groups where plasma neutralization activities increased with time since infection [31,32]. All four of these subjects showed effective neutralization of at least the lab strains NL4-3 and BAL, with patients #4 and #10 showing an even more robust neutralization of the viral isolates AC10 and SVBP16 in some of the samples (Figure 4, left hand panels). As this activity was mainly seen in samples at the beginning of the treatment interruption (i.e. on-treatment) it may reflect the presence of residual antiretroviral drugs, particularly Efavirenz, which has been described to have a long plasma half-life [33-37]. Indeed, these two patients were the only ones who were receiving Efavirenz as part of their antiretroviral treatment (Table 1) and the neutralization assays were thus repeated after purifying IgG fractions from plasma. Both, the IgG purified fraction and the negative elute fraction were tested in neutralization assays against NL4-3 and VSV-HIV-pseudotyped virus. As suspected, the IgG-depleted elute (used as a 1/60 dilution) was able to inhibit viral replication completely (100% of neutralization activity) for NL4-3 virus. The purified fraction tested at 200 ug/ml reached around the 80% of neutralization for NL4-3 virus in these two patients. Thus, the strong neutralization activity seen in unpurified plasma was likely largely due to residual antiretroviral in samples taken on the day of treatment stop. In contrast, for patients #2 and #7, the neutralization capacity was contained in the purified IgG fraction (80% of neutralization capacity against NL4-3 when tested at 200 ug/ml) and not in the IgG-depleted elute (16%-54% when tested at a 1/60 dilution). Of note, all neutralization activities observed in the purified IgG fractions were HIV-specific, since no neutralization was seen when using VSV-peudotyped NL4-3 virus as negative control (data not shown).

## Discussion

The presence of HIV-specific neutralizing antibodies has been associated in several studies with at least partial viral control after treatment interruption [25,27]. However, the frequency at which infected individuals are able to regain effective nAb activity after STI varies widely and may be impacted by the general and B cell-specific immune status of the subject undergoing STI. To our knowledge, the present report is the first study that specifically addresses the effects of STI on the frequency of antibody secreting cells and their production of nAb. In the case of Influenza vaccination in healthy, HIV uninfected, individuals the exposure to vaccine-derived viral antigen has been shown to lead to a rapid expansion of ASC that also produced high titers of nAb [38]. We hypothesized that STI in HIV infection could mimic such antigen exposure and thus aimed to use existing samples from a larger, past STI trial to characterize the effects of STI on ASC frequency and nAb activity. The use of retrospective samples was needed as the STI/self-vaccination approaches have not been holding their initial promise to lead to effective in vivo viral control and are nowadays generally limited to “analytical STI” in the setting of therapeutic interventions. With the use of retrospective samples however, the present study was severely limited to the existing time points and patients from which specimens were collected.

In previous studies, with immunizations using a *Salmonella typhi* oral vaccine, ASC were found to appear in the peripheral blood a few days after vaccination, reaching the highest levels on day 7 and were again undetectable 2 weeks after vaccination [39]. The same kinetics were also seen in the study by Wrammert et al. using influenza vaccination where influenza-specific ASCs in peripheral blood peaked at day 7 post immunization, returning to basal levels by day 14 after vaccination [38]. The trial, from which samples were available for the present study, sampled peripheral blood after 2 weeks of treatment interruption, putting our analyses potentially on the far end of ASC reactivation. Probably, the peak in ASC may not be as early for instance as after a (Flu) vaccination where antigen is given at once and generally in healthy individuals. Furthermore, the individuals undergoing treatment interruptions are HIV infected and at least partly immune compromised subjects, for which rapid and strong changes in ASC may actually come rather as a surprise. Despite this concern, our data clearly indicate that ASC are expanded after re-exposure to HIV antigens, particularly in subjects with clinically detectable (>1100 copies/ml) viral rebounds within these two weeks. We can however not exclude that subjects with subclinical viral rebound also drove their ASC populations; possibly to a lower extend and which may not be detectable anymore at 14 days post treatment interruption. On the other hand, and in contrast to the immediate availability of antigen upon for instance Flu vaccination, re-exposure to sufficient quantities of the autologous HIV may require a few days of viral replication (and reduction of antiretroviral drugs) to reach effective stimulatory levels and could thus possibly delay their emergence in the peripheral blood. Nevertheless, the quite rapid kinetics of ASC fluctuations described in the vaccination studies above as well as HIV-related STI setting are further documented by the return of ASC levels to basal values within only the 4 weeks of on-treatment cycles. While these kinetics and our data presented here suggest that these fluctuations are driven by the expansion of HIV-specific B cells, our data can not document this directly since our assays were not geared towards the detection of HIV-specific ASC. However, in previous studies by Doria-Rose et al., B cells with a CD3^-^CD19^+^CD20^-/low^CD27^hi^CD38^hi^ plasmablast phenotype were indeed increased in HIV infection and accounted for the vast majority of HIV-specific ASC (up to 92% of gp-120-specific ASC) [13]. This, together with the stable total IgG levels seen in most of our patients during on-off treatment cycles strongly suggests that STI and subsequent viral reactivation drive preferentially the expansion of HIV-specific ASC. Given the B cell phenotype and the rapid kinetics, it is also likely that the increased B cell responses were due to a reactivation of pre-existing memory B cells rather than stimulation of newly generated ASC.

When assessing gp120 specific Ab production upon STI, we did not observe a correlation between ASC frequencies and anti-gp120 IgG levels in the plasma. As all patients showed detectable anti-gp120 IgG levels in the baseline sample, only mild increases in their concentration upon STI may have been masked by anti-gp120 IgG produced by long-term memory plasma cells that continuously produce antibodies without the need of antigen exposure [40]. Alternatively, IgG of other HIV-specificities not assessed here (including gp41 specific responses) could have been produced, making us miss a potential correlation between increases in HIV-specific ASC levels and total anti-viral IgG. The same limitation applies when the gp120 levels were compared to neutralization activities. After controlling for residual antiretroviral drugs in the plasma at times when treatment was interrupted, only the two subjects with highest gp120 IgG levels showed neutralization activity. It remains thus unclear whether additional specificities may have contributed to this activity aside from the gp120 specific cells and what the fraction of non-neutralizing IgG among the anti-gp120 fraction was [13,41-43].

Overall, 20% (2/10) of the patients showed neutralizing activity in purified IgG fractions. These subjects were among the 5 individuals who showed robust reactivation of their virus during all STI cycles and who had a reduced CD4 T cell count at the start of first STI (median CD4 count 801/ul) compared to the five subjects that did not show consistent viral reactivation (median CD4 count 1122/ul). Of note, the fluctuations in neutralization activities are not uniformly paralleled in these two subjects, possibly reflecting variable parameters that govern their kinetics, including growth kinetics of autologous virus, existing T-helper cell immunity (especially follicular helper CD4 T cell responses that are needed for B cell differentiation into plasma cells) and the presence of memory B cells in lymph nodes [44-47]. In addition, the sustained presence of memory B cell populations with low levels of Foxo3a and TRAIL-mediated apoptosis has been associated with relative viral control and may have further impacted ASC expansion upon STI [48]. Regardless of these limitations, STI may offer a window of opportunity to gain access to samples with highly enriched frequencies of HIV-specific ASC in at least some individuals, especially with the identification of additional parameters that can predict their presence reliably.

## Conclusions

In summary, our results suggest that antigen re-exposure after STI can lead to substantial increases in the frequencies of ASC and, at least in some patients, this increase is associated with the enhancement of HIV-specific IgG-mediated neutralizing activities. While additional factors that limit such a recovery in neutralizing activity to few individuals remain to be identified, analytical STI in therapeutic vaccine trials or the screening of extensive sample banks from past STI trials may offer opportunities to isolate ASC producing nAb at significantly elevated levels, facilitating the identification and characterization of new nAb against HIV, and thereby providing crucial information for future HIV vaccine design.

## Competing interests

The authors declare that they have no competing interests.

## Authors’ contributions

CB conceived the experiments, participated in its design and collaborated to draft and to write the manuscript. ALL designed the experiments, carried out the flow cytometry, and the IgG purifications assays, performed the statistical analysis, analyzed the data and wrote the manuscript. JC carried out the ELISA assays and participated in the data interpretation. JB participated in experiments design, data discussion and manuscript revision. EY, VS, BM and BC helped in data discussion and manuscript revision. LR helped in selecting the samples and in drafting the manuscript. SM and EG carried out the neutralization assays and helped in their analysis. All authors read and approved the final manuscript.
